# Assessing the Independent Contribution of Maternal Educational Expectations to Children’s Educational Attainment in Early Adulthood: A Propensity Score Matching Analysis

**DOI:** 10.1371/journal.pone.0119638

**Published:** 2015-03-24

**Authors:** Jean Baptiste Pingault, Sylvana M. Côté, Amélie Petitclerc, Frank Vitaro, Richard E. Tremblay

**Affiliations:** 1 Division of Psychology and Language Sciences, University College London, London, United Kingdom; 2 Research Unit on Children’s Psychosocial Maladjustment, University of Montreal and Sainte–Justine Hospital, Montreal, Quebec, Canada; 3 International Laboratory for Child and Adolescent Mental Health Development, University of Montreal, Montreal, Quebec, Canada; 4 Institute of Genetic, Neurobiological and Social Foundations of Child Development, Tomsk State University, Tomsk, Russian Federation; 5 Teachers College, Columbia University, New York, New York, United States of America; 6 School of Public Health, Physiotherapy and Population Science, University College Dublin, Dublin, Ireland; 7 Department of Pediatrics, University of Montreal, Montreal, Quebec, Canada; 8 Departments of Psychiatry, University of Montreal, Montreal, Quebec, Canada; 9 Departments of Psychology, University of Montreal, Montreal, Quebec, Canada; Hospital Universitário, BRAZIL

## Abstract

**Background:**

Parental educational expectations have been associated with children’s educational attainment in a number of long-term longitudinal studies, but whether this relationship is causal has long been debated. The aims of this prospective study were twofold: 1) test whether low maternal educational expectations contributed to failure to graduate from high school; and 2) compare the results obtained using different strategies for accounting for confounding variables (i.e. multivariate regression and propensity score matching).

**Methodology/Principal Findings:**

The study sample included 1,279 participants from the Quebec Longitudinal Study of Kindergarten Children. Maternal educational expectations were assessed when the participants were aged 12 years. High school graduation – measuring educational attainment – was determined through the Quebec Ministry of Education when the participants were aged 22–23 years. Findings show that when using the most common statistical approach (i.e. multivariate regressions to adjust for a restricted set of potential confounders) the contribution of low maternal educational expectations to failure to graduate from high school was statistically significant. However, when using propensity score matching, the contribution of maternal expectations was reduced and remained statistically significant only for males.

**Conclusions/Significance:**

The results of this study are consistent with the possibility that the contribution of parental expectations to educational attainment is overestimated in the available literature. This may be explained by the use of a restricted range of potential confounding variables as well as the dearth of studies using appropriate statistical techniques and study designs in order to minimize confounding. Each of these techniques and designs, including propensity score matching, has its strengths and limitations: A more comprehensive understanding of the causal role of parental expectations will stem from a convergence of findings from studies using different techniques and designs.

## Introduction

### Educational expectations’ role in educational attainment

Parental educational expectations have been associated with children’s educational attainment in a number of long-term longitudinal studies [1–7]. All but one [4] found a positive statistically significant contribution of expectations to educational outcomes. Whether this consistent relationship between parental expectations and children’s educational attainment is causal has long been debated (see [8], and [9]). The issue of causality is of paramount importance in order to determine if expectations are valuable targets for intervention. Although we cannot draw strict causal inferences from observational studies, several authors have recently emphasized the need to build on research designs and "statistical innovations that allow for stronger causal inference" [10] as a crucial complement to experimental studies [11]. The purpose of this study was to build on one such tool, propensity score matching, to examine the relationship between low maternal educational expectations, assessed when the children were aged 12 years, to high school graduation 10 years later. To simplify, the term propensity score matching is used here to refer to the large family of methods used to equate the distribution of covariates in the treated and control groups.

### Educational expectations and selection bias

Selection bias is an important problem when trying to assess the contribution of maternal expectations to the child’s educational attainment. Maternal expectations are far from being distributed at random in the population. For instance, maternal expectations are influenced by social characteristics such as the mother’s own level of education [12,13]. In turn, maternal education is a key predictor of the child’s educational attainment [6,13,14]. Mothers with higher levels of education are thus likely to have higher educational expectations for their children, and their children are more likely to succeed in school. Hence, maternal expectations may constitute a by-product of maternal education and, consequently, retains no causal role in predicting children’s educational attainment. Failing to take into account maternal education would thus seriously bias the estimation of the contribution of maternal expectations to educational attainment. A number of variables are likely to play such a confounding role. Socioeconomic characteristics of the family strongly predict both expectations and educational outcomes [12–19]. Similarly, family structure or changes in the family (e.g. intact versus non-intact family, house moving) and parenting variables may also represent a source of bias [12,14,19,20]. It is also important to account for two sets of child characteristics highly correlated with educational outcomes and maternal expectations such as child’s school performance [3,9,14,21,22] or children’s behaviors, in particular inattention [6,23,24].

To assess the long-term contribution of parental expectations to a range of education outcomes, several studies utilized longitudinal designs controlling for a restricted number of the confounding variables outlined above [2,3,5–7]. All of these studies [2,3,5–7] reported a statistically significant long-term contribution of parental expectations to educational outcomes. However, the contribution of parental expectations to educational outcomes may have been overestimated as important control variables were omitted.

Two studies stand out [1,4] as they controlled for many more potential confounders. Alexander et al. [1] still detected a statistically significant beneficial contribution of parental expectations to school outcomes. Ou and Reynolds [4], who included the largest range of variables, examined the contribution of parental expectations, measured at 10 years, in a series of models with different blocks of predictors and several high school outcomes, including high school graduation. Parental expectations made a statistically significant contribution when first introduced in their models, along with control variables and blocks of predictors measured mostly before age 10 years. However, they became non-significant after the inclusion of additional variables mostly measured at age 10 years or later. Therefore, the non-significant contribution of parental expectations in the final model may reflect mediational processes (i.e. there is a true causal chain from parental expectations to attainment but through later intervening variables). In sum, the contribution of parental expectations to educational outcomes may have been overestimated since most studies included a limited number of control variables; yet, in the study with the largest number of control variables [4], their contribution may have been underestimated because of the inclusion of later intervening variables.

### A propensity score approach

As outlined above, including only a restricted set of potential confounders may overestimate effects. However, including a large set of potential confounders in multivariate regressions (as in [1], and [4]) is also problematic. First, as emphasized by Rubin [25]: “regression analysis cannot reliably adjust for differences in observed covariates when there are substantial differences in the distribution of these covariates in the two groups” (for instance substantially different mean levels of income in a high expectation group versus a low expectation group). When regression approaches cannot remove all or nearly all the bias, alternative strategies such as propensity score matching can be used [25]. Second, propensity score matching also deals with additional problems encountered in regression models: multicollinearity, modeling choices and specifications as, for instance, choosing a linear model over a quadratic one [26]. Multicollinearity in particular may be an issue when using a large number of confounders as covariates in multivariate regressions.

Following is an illustration of propensity score matching, using income as an example of confounding variable. Children exposed to low maternal educational expectations differ from those exposed to high maternal educational expectations as they tend to come from families with a lower income [12]. Therefore, the difference in graduation failure between the low and the high expectation groups may come from the expectations themselves but also from the existing differences in mean income between the two groups. Matching techniques [26,27] enable the selection, for each participant in the low expectation group, of a matched participant in the high expectation group with a very similar family income. After matching, no difference in mean income should remain between the two groups; because the mean income is the same in the two groups, income can no longer introduce a bias in the estimation of the effect of maternal educational expectations on educational attainment. *Propensity score matching* is a generalization of this procedure to the multivariate case: after matching, the two groups should be balanced on a set of confounders (i.e. for each confounder, the mean should be very similar in the two groups). The *propensity score*, generated for each participant and used to match them, is the probability of belonging to the exposed group conditional on a set of confounders. For instance, in this study, children in the low maternal expectation group have a high probability of belonging to this group given a set of confounders (e.g. lower income and maternal education); however, in the high expectation group, some participants also have a high probability of belonging to the low expectation group but, nevertheless, belong to the high expectation group (e.g., children whose mothers have low income and education, yet have high educational expectations for their children). The matching procedure aims to reduce the *distance* between the two groups, i.e. the propensity score standardized mean difference between the two groups. The standardized mean difference must be reduced for the propensity score but also for each confounder. As a general rule, it is important that the confounders included in the propensity score are measured before exposure [25,26]. For instance, in the present case, a variable measured after, (i.e. school achievement at a later period) may already have been influenced by low maternal expectations, so that the level of this variable may be legitimately lower in the group with low maternal expectations.

In the present study, we implemented the approaches used in the literature (i.e. small or large set of confounders) to compare their results with those obtained using propensity score matching. The aim was two-fold: 1) test whether we could replicate the results found in most previous studies (i.e. the significant association between educational expectations and educational outcomes); 2) test if the use of propensity score matching changed the results.

Finally, the role of expectations may not be equal across the entire population. In particular, sex may moderate the association between expectations and educational attainment. Several authors have highlighted the need to consider sex differences more systematically in studies on expectations and their consequences [3,13]. Whether the contribution of parental expectations to educational outcomes differs between males and females, has been examined by very few studies [28,29] with mixed results. We thus tested the interaction between maternal expectations and sex.

## Methods

The study has been approved by the University of Montreal Ethics Committee. After complete description of the study, written consent was obtained from the mothers at each wave of data collection (including consent regarding teachers’ reports).

### Participants

The study sample included 1,279 participants belonging to the Quebec Longitudinal Study of Kindergarten Children (presented in detail in [30]). In this cohort, 3,007 children were first assessed in 1986–1987 when they were in kindergarten in Quebec’s French-speaking public schools (Canada) and followed thereafter. A total of 1,776 participants had data for both maternal educational expectations – assessed when the children were 12 years – and educational attainment at age 22–23 years. We selected the 1,279 participants with low and high maternal expectations (see details below). Table 1 presents the demographic characteristics of the study sample.

**Table 1:** Demographic Characteristics of the Study Sample.

	*M* (or %)	*SD*
Sex of the child (males)	50.3	-
Maternal education	12.15	2.76
Paternal education	12.38	3.60
Occupational socioeconomic index (mother)	44.57	13.16
Occupational socioeconomic index (father)	44.80	15.36
Age mother (at child’s birth)	27.20	4.52
Age father (at child’s birth)	29.61	5.28
Intact family (at 6 years)	13.1	-

### Measures

#### Outcome variable

In 2003, when the participants were aged 22–23 years old, information about high school graduation was obtained through the Quebec Ministry of Education in 2003 with permission from the Québec Data Access Board. The measure differentiated between participants who had a high school diploma (coded zero) and participants who did not (coded one, which included those who dropped out and those who were still attending school).

#### Independent/exposure variable

When the child was 12 years old, the mother answered the following question: At what level do you want your child to finish studying? The answer was (1) to finish high school or a lower level (N = 211); (2) to terminate Cegep (N = 497); and (3) to obtain a university degree (N = 1068). The colleges (Cegep) provide a program that is a requirement for entry to university. Students who complete high school (after 11 years of schooling) must complete two years of the colleges and then proceed to university for completion of their program.

Although matching techniques in multi-group cases are being developed (called nonbipartite matching), most existing matching methods in the literature focus on two groups (called bipartite matching) [31] and software applications such methods [32] have richer features (e.g. matching algorithms and options). We thus only considered children whose mother had low or high expectations and discarded children whose mother had intermediate expectations, leading to the study sample of 1,279 children.

Maternal educational expectations represent how far mothers *think* or expect their child will go, whereas maternal educational aspirations represent how far mothers *want* their child to study. Although distinct at the conceptual level and, somewhat, at the empirical level [9,33], expectations and aspirations are highly correlated and the two concepts have often been used as proxies for one another [34]. We adopted this approach and used our question about what mothers want for their child educational attainment as a proxy of maternal expectations.

#### Potential confounders

Multicollinearity is less of a problem when using propensity score matching than when using multivariate regression [26]. It is thus possible to be more liberal in the inclusion of potential confounders and, therefore, reduce the potential bias due to unobserved confounding variables. We selected variables that represented a well-documented source of bias as well as variables that could represent a potential source of bias even in the absence of extensive literature on the topic. We then tested whether these variables were associated with the predictor *and/or* the outcome [27]. A total of 60 potential confounders were tested for inclusion in the propensity score. 54 variables were retained and are listed in Table 2.

**Table 2:** Balance in Covariates Before and After Matching between the Low Maternal Expectations Group and the High Maternal Expectations Group.

	Means			Standardized Mean Differences	
	Low Expectations Group (N = 211)	High Expectations Group (N = 1068)	High Expectations *After Matching* (N = 124)	Before Matching	*After Matching*
Distance	0.46	0.11	0.45	1.320	0.037
**Family characteristics and parenting**					
Maternal education^1^	10.40	12.47	10.40	−0.974	−0.001
Paternal education	9.88	12.73	9.85	−1.148	0.014
Occupational socioeconomic index (Mothers)	35.07	42.64	34.61	−0.838	0.051
Occupational socioeconomic index (Fathers)	36.43	45.87	36.40	−1.207	0.005
Income	6.14	8.82	6.12	−1.004	0.010
Age mother (at child’s birth)	26.01	27.44	25.63	−0.297	0.080
Age father (at child’s birth)	28.97	29.65	28.74	−0.134	0.045
Number of children	2.59	2.24	2.51	0.295	0.066
Non-intact family	0.22	0.23	0.25	−0.012	−0.075
Moving house	0.43	0.36	0.48	0.141	−0.088
Falender: stimulation	-1.41	-1.60	-1.70	0.054	0.081
Falender: pleasure	13.39	14.29	13.84	−0.143	−0.071
Falender: authority	-8.59	-8.01	-9.09	−0.125	0.106
**Child individual characteristics and medical record**					
Sex	0.56	0.49	0.56	0.148	−0.001
Physical abnormalities	0.04	0.02	0.03	0.060	0.039
Dentist	1.39	1.32	1.31	0.115	0.137
General practitioner or paediatrician	1.10	1.17	0.99	−0.099	0.173
Speech therapist	0.30	0.17	0.27	0.295	0.071
Psychologist	0.25	0.11	0.17	0.311	0.164
Psychiatrist	0.05	0.02	0.02	0.140	0.123
Special needs teacher	0.31	0.13	0.27	0.386	0.098
**Child temperament. behaviors and emotions**					
DOTS: Attention	5.95	7.02	6.29	−0.359	−0.113
DOTS: Rhythmicity	5.99	6.51	5.93	−0.251	0.028
DOTS : Reactivity	3.00	2.78	2.85	0.141	0.095
DOTS: Activity	0.44	0.34	0.46	0.201	−0.042
Inattention (Teachers) ^1^	3.73	1.88	3.44	0.922	0.146
Hyperactivity (Teachers)	1.02	0.61	0.97	0.381	0.047
Physical Aggression (Teachers)	0.75	0.44	0.77	0.284	−0.018
Opposition (Teachers)	1.87	1.30	1.78	0.323	0.048
Anxiety (Teachers)	2.45	1.77	2.33	0.475	0.086
Inattention (Mothers)	3.52	2.39	3.36	0.654	0.093
Hyperactivity (Mothers)	1.79	1.39	1.73	0.356	0.048
Physical Aggression (Mothers)	1.14	0.75	1.00	0.355	0.125
Opposition (Mothers)	3.27	2.81	3.07	0.271	0.120
Anxiety (Mothers)	3.58	3.22	3.48	0.214	0.059
Difficulties: behavioral (Teachers)	0.56	0.29	0.57	0.541	−0.008
Difficulties: relational (Teachers)	0.46	0.30	0.44	0.319	0.044
Difficulties: behavioral (Mothers)	0.41	0.25	0.41	0.329	0.009
Difficulties: relational (Mothers)	0.28	0.17	0.25	0.241	0.062
Emotional troubles (Mothers)	0.48	0.38	0.50	0.208	−0.030
Get along with family	1.84	1.68	1.78	0.236	0.096
**Child academic record**					
Grade retention^2^	0.34	0.08	0.34	0.539	0.00
Reading	2.54	3.59	2.59	−1.159	−0.056
Writing	2.45	3.45	2.47	−1.081	−0.024
Maths	2.76	3.63	2.86	−1.013	−0.114
Global performance^1^	2.58	3.56	2.64	−1.144	−0.069
Get along with teachers	1.59	1.35	1.56	0.359	0.049
Like school	2.18	1.81	2.15	0.487	0.042
Difficulties: academic performance (Teachers)	0.64	0.24	0.64	0.835	0.006
Difficulties: motivation (Teachers)	0.68	0.29	0.67	0.833	0.018
Difficulties: diligence (Teachers)	0.16	0.08	0.21	0.232	−0.142
Difficulties: academic performance (Mothers)	0.47	0.19	0.44	0.547	0.046
Difficulties: motivation (Mothers)	0.60	0.27	0.59	0.665	0.024
Difficulties: diligence (Mothers)	0.12	0.06	0.13	0.198	−0.015
Average Absolute Standardized Mean Differences^3^	-	-	-	0.438	0.063

*Note*. Each standardized mean difference is obtained by: mean in the low expectation group minus mean in the high expectation group, divided by the standard deviation in the low expectation group.

^1^Mahalanobis distance used.

^2^Exact matching used.

^3^The average absolute standardized mean difference is the average of the absolute values of standardized mean differences for all covariates.

#### Family characteristics and parenting

Maternal and paternal education were measured by the number of years of schooling assessed when the child was 6–7 years old. *Occupational socioeconomic index* of the mother and the father were assessed when the child was aged 6 years, based on Blishen et al. [35]. *Annual income* of the whole family was assessed when the child was 7 years old and divided into categories (from 1 to 13) of $5,000 (Canadian), with category 1 being <$5,000 and category 13 being >$60,000. *Age of the mother* and *of the father* when the target child was born, as well as the *number of children* in the family (assessed when the target child was 9 years). *Intact family* was coded 1 when the mother reported at least once that the child lived in a non-intact or step-family between ages 6 and 11 years (reporting was available every year); it was coded 0 otherwise. *Residential move*: a similar coding was adopted to reflect whether the mother reported a residential move between ages 6 and 11 years. Mothers completed a French version of a parent child-rearing attitude questionnaire when the child was age 6 and 7 years [36]. Mothers responded to each item on a 4-point scale ranging from “very false” to “very true”. Three dimensions were assessed: *Pleasure* (18 items, alphas for the two years: 0.81, 0.83), *Stimulation* (12 items, alphas: 0.51, 0.51), *and Discipline* (16 items, alphas: .63, .60). The scores were averaged across the two years.

#### Child individual characteristics and medical records

The *sex* of the child was coded 1 for boys and 0 for girls. When the child was 11 years, the mother was asked whether the child had *physical abnormalities* when he/she was born (0 = no; 1 = yes). Each year between 6 and 11 years, the mother was asked if the child had gone to a *general practitioner or pediatrician*: “Never” (= 0), “one or two times” (= 1), “several times” (= 2), “regularly” (= 3). The variable was averaged over the 6 years. The same question was asked for visits to a *dentist* and was coded in the same way. The same question was also asked for the following four specialties: *speech therapist*, *psychologist*, *psychiatrist* and *special needs teacher*. Few mothers reported that their child had consulted such specialists between 6 and 11 years; these 4 variables were thus recoded 1 when the mother ever reported that her child had consulted and 0 otherwise.

#### Child temperament, behaviors and emotions

At age 6 years, mothers rated the child’s temperament with a French version of the Dimensions of Temperament Survey (DOTS) [37], with each item coded 0 for “rather false” and 1 for “rather true”. The original scale score was used for the first four dimensions: *attention span* (11 items, alpha: .80); *adaptability* (6 items, alpha: .67); *rhythmicity* (8 items, alpha: .70); *reactivity* (6 items, alpha: .59). Few children obtained a non-null score on the *activity* scale (3 items) so we recoded it with 0 when only zero scores were reported and 1 when a non-null score was reported.

A different teacher each year rated behavioral characteristics of the child between ages 6 and 11 years, with the Social Behavior Questionnaire (SBQ [38]). Each item was rated on a three-point scale (0–2, from ‘never applies’ to ‘frequently applies’). The five scores corresponding to the five behaviors were averaged between ages 6 and 11 years. Rated behaviors were: *inattention* (4 items, alphas range: .85-.90), *hyperactivity* (2 items, alphas range: .83-.88), *physical aggression* (3 items, alphas range: .81-.86), *opposition* (5 items, alphas range: .79-.84), *anxiety* (5 items, alphas range: .70-.75). Mothers also rated behavioral characteristics of the child using the same SBQ items at the same ages. Rated behaviors were: *inattention* (alphas range: .70-.82), *hyperactivity* (alphas range: .76-.79), *physical aggression* (alphas range: .57-.67), *opposition* (alphas range: .62-.69), *anxiety* (alphas range: .60-.67).

Both mothers and teachers reported each year between the 1^st^ and the 5^th^ grade if the child had *behavioral or emotional difficulties* as well as *relational difficulties* (yes = 1; no = 0). The four resulting variables were coded similarly: 1 when the informant answered yes at least once during the 5 years; 0 when the informant answered a no at least once but no yes. Mothers also answered a similar question regarding whether the child had *emotional troubles* during the same period; this variable was coded in the same way. Mothers also rated from 1 (“very well”) to 5 (“not well at all”) how well the child was *getting along with his/her family* from 7 to 11 years. The score was averaged across the 5 years.

#### Child academic background

Teachers rated child achievement each year from the age of 7 to 11 years (the kindergarten assessment was not available) with three questions on child’s performances in *writing*, *reading* and *mathematics*. Each question was coded on a 5-point scale, from markedly below the mean (1) to markedly above the mean (5). Each of these 3 scores was averaged across the 5 years. Teachers also rated the *global performance* of the child on the same 5-point scale. From the 2^nd^ to the 5^th^ grade teachers reported grade retention. The binary variable was coded 1 when any retention was reported between these grades. Both mothers and teachers rated each year between the 1^st^ and the 5^th^ grade if the child was having difficulties in *academic performance*; *motivation; and diligence* (yes = 1; no = 0). The six resulting variables were coded similarly: 1 when the informant answered yes at least once during the 5 years; 0 when the informant answered ‘no’ at least once and never answered yes. Mothers rated from 1 (“likes a lot”) to 4 (“does not like at all”) if the child *liked school* at ages 10 and 11 years. The score was averaged across the 2 years. Mothers rated from 1 (“very well”) to 5 (“not well at all”) how well the child was *getting along with his/her teacher* from 7 to 11 years. The score was averaged across the 5 years.

Finally, 6 variables were tested but not included in the propensity score matching procedure (see Data analysis for a rationale): birth weight, whether the child was in an incubator after birth, whether the child was hospitalized at age 7 or 8 years, the adaptation score of the aforementioned DOTS scale, and whether the child saw an occupational therapist or another doctor besides all the previously mentioned specialists.

### Data analysis

#### Propensity score matching

We tested the bivariate relationships between each potential confounder and maternal educational expectations *or* high school graduation, (with logistic regressions). The aforementioned 54 variables were significantly associated (p < .05) with either maternal educational expectations or high school graduation, or both, and were therefore retained to create the propensity score. Several matching options should be tested to find the one that best reduces the distance between the two groups [26,27]. We tested nearest neighbor and full matching because they use different algorithms to match participants. *Nearest neighbor matching*: for each participant in the exposed group, the participant with the closest propensity score (i.e. the smallest distance) is found in the control group. Several options can be used and combined including: 1) using several matched controls for every exposed participant; 2) exact matching for important categorical variables, leading for example to exactly the same proportion of children having grade retention between the two groups; 3) the Mahalanobis distance measure which enables closer matching for a small number of important continuous variables (e.g. maternal education); 4) matching with replacement i.e. a given control can be matched to several exposed participants; 5) a discard option for participants outside common support, i.e. the overlapping range of propensity scores between the two groups [26,27,32]. *Full matching* [39] is a technique which enables a flexible matching: within subclasses, an exposed participant can be matched with several controls but, also, several exposed participants can be matched with one control. Restrictions can be specified to avoid an excessive number of controls being matched with one exposed participant and vice versa. Although full matching can be used to keep all controls, it is also possible to specify how many controls should be kept in the matching procedure (e.g. use 2 participants with high expectations to match each participant with low expectations) and the aforementioned discard option is also available.

Assessing the balance between the two groups after matching can be achieved with the standardized mean differences (also referred as “standardized bias” or “effect sizes”) for the propensity score as well as for each of the confounders included in the model [27]. While recognizing that there is no consensus cut-off, some authors suggested using a standardized mean difference smaller than 0.05 after matching [40]. Other authors suggested to analyze standardized mean differences as effect sizes (i.e. 0.2 considered small, 0.5 medium and 0.8 large) and compare them before and after matching for all variables in order to assess the success of the matching procedure [41].

#### Multiple imputation

The percentage of missing data was less that 10.0% for most of the 60 potential confounders, with the following exceptions: physical abnormalities (13.1); intact family (14.2); incubator (14.4); birth weight (16.7); number of children in the family when the child was 9 years (49.9). Given the large number of potential confounding variables, an analysis restricted to complete cases would have yielded an important bias. We thus used multivariate imputation by chained equations and imputed 30 data sets [42,43]

### Contributions of low maternal expectations to failure to graduate

#### Regression estimates

We present both odds ratios (ORs) and risk ratios (RRs) as measures of risk. Available studies on educational expectations with dichotomous outcomes used ORs. However, for common outcomes, when a risk ratio is greater than 1, the corresponding odds ratio will be further from 1, which can be misinterpreted as a greater risk [44]. For instance, when an outcome is rare, e.g. 1%, ORs and RRs agree; however, serious distortions start even at 10% [44]. ORs are thus a potential source of bias to estimate the effect of low expectations on a common outcome such as graduation failure. Furthermore, contrary to RRs, ORs are not collapsible, meaning that the ORs will change even when adjusting for a variable that is not a confounder, complicating their interpretation [44].

To enable comparison with available literature, we present three sets of regression estimates of the contribution of low expectations to failure to graduate from high school:

1. Unadjusted estimates with no confounding variable;
2. Adjusted estimates, using a small set of important confounding variables. We selected a small set of variables that have been controlled for in most of the available studies (see introduction), regarding the family’s socioeconomic characteristics, the child’s previous achievement and the child’s sex; we also added variables regarding family structure as well as the child’s inattention, which has been shown to be an important behavioral predictor of educational outcomes [12,23,24]. Variables included in this second model are: income, maternal education, teacher rated global academic performance, grade retention, child’s sex, moving house, intact family, teacher rated inattention. These estimates were computed using the multiply-imputed data sets.
3. Fully adjusted estimates with all the variables included in the propensity score matching. These estimates were computed using the multiply-imputed data sets.

#### Propensity score matching estimates

Each matching procedure was repeated across the 30 imputed data sets and the one that best balanced the low and high expectation groups was retained. Then, the estimates of the contribution of low maternal educational expectations to high school graduation failure were combined across data sets [32,45,46]. Finally, in order to verify the robustness of the results, we conducted complementary analyses which are presented under the Complementary analyses heading in the Results section.

Analyses were conducted within the R software [47] using packages *mice* to generate imputed data sets [43], *MatchIt* for propensity score analyses [32], and *Zelig* for final model estimations using the imputed data sets [46].

## Results

### Propensity score matching

We tested the different combinations of matching options presented in the method section. All options matching more than one control to every exposed participant and not using replacement (i.e. not allowing one high expectation participant to be used several times to match one low expectation participant) did not yield satisfactory results. Nearest neighbor matching, with a ratio of 1 to 1, allowing for replacement led to the lowest distance between the two groups, very close to zero (0.003). However, the matching was not optimal for variables that have been demonstrated to be crucial confounders in the prediction of educational outcomes, in particular, maternal education, teacher rated global academic performance and teacher rated inattention [26]. We thus used Mahalanobis distance for these three variables as well as exact matching on grade retention (which helped in the matching). With these specifications, the distance on these variables was further reduced, in particular for maternal education, and we obtained an overall distance of 0.037. We therefore chose this option. Given that replacement was possible, an average (over the 30 imputations) of 124 participants with high maternal expectations was matched to the 211 participants with low maternal expectations.

Table 2 shows the means for each variable: (1) in the exposed group (low expectations), (2) in the control group before matching (high expectations) and (3) in the control group after matching (matched participants with high expectations); it also shows the standardized mean differences before and after matching. For instance, before matching, mothers in the low expectation group had 10.4 years of education in average, two years fewer than in the high expectations group (12.5, for a standardized mean difference of −0.97); after matching, mothers of the matched participants in the high expectations group also had an average education of 10.4 years (for a standardized mean difference of 0.00). Standardized mean differences between the two groups before matching were large (≥.80) for 12 variables including socioeconomic variables, teacher rated school performance and teacher rated inattention. Most of the other differences were medium (≥0.50) or small size (≥0.20). After matching, none of the standardized mean differences reached the small effect size threshold; absolute values were all between 0.00 and 0.17. Therefore, although some variables were above the more stringent 0.05 cut-off [40], the matching procedure considerably reduced the bias. Finally, the differences in empirical distributions of variables in the two groups were greatly reduced after matching, in particular for important confounding variables identified in the literature (i.e. maternal education, teacher rated global academic performance and teacher rated inattention).

### Effect of exposure to low maternal expectations on failure to graduate

#### Regression estimates

Table 3 presents ORs and RRs for the unadjusted, adjusted and fully adjusted regression models. A total of 65.9% participants failed to graduate from high school by age 22–23 years in the low maternal expectations group against only 21.4% in the high expectation group, corresponding to an unadjusted RR of 3.07 (p <.001). Graduation failure was thus 3 times more frequent in the low expectation group. In the adjusted model including a restricted set of key confounding variables (income, maternal education, teacher rated global academic performance, grade retention, child’s sex, moving house, intact family, teacher rated inattention), the role of maternal expectations was greatly reduced but was still statistically significant (RR: 1.26, p = .008). In the fully adjusted model, the RR was further reduced and was only marginally significant (RR: 1.20, p = .059). The interaction between expectations and sex was positive and marginally significant in the unadjusted model; it was positive but not statistically significant in the adjusted and fully adjusted models. Of note is that ORs not only were much larger than RR (e.g. unadjusted OR of 7.07 against RR of 3.07) but also led to a different conclusion, as all ORs were statistically significant, including for the fully adjusted model (OR: 1.97, p = .004). Thus, the sole consideration of ORs resulting from multivariate regressions would be that low expectations make a highly statistically significant contribution to graduation failure.

**Table 3:** Contribution of Maternal Low Expectations to Failure to Graduate from High-School in Multivariate Regression Models.

	Odds Ratios				Risk Ratios			
	OR	B	Se	P value	RR	B	Se	P value
Unadjusted	7.07	1.96	0.16	<.001	3.07	1.12	0.08	<.001
Adjusted	2.12	0.75	0.21	<.001	1.26	0.23	0.09	.008
Fully adjusted	1.97	0.68	0.24	.004	1.20	0.18	0.09	.059

*Note*. The coefficients presented in the first four columns were obtained from logistic regressions: Odds Ratios (OR), estimate (B), standard error (Se), and p values. Poisson regressions with robust sandwich estimator of variance were used to obtain the estimates in the last four columns, including unadjusted and adjusted risk ratios (RR) [44]. OR and RR are exponential of their respective estimate (B). In the unadjusted model, no confounder was entered. In the adjusted model, 8 essential confounders were considered (see Method section). In the fully adjusted model, all 56 variables were included as confounders.

#### Propensity score matching estimates

**Fig 1:**
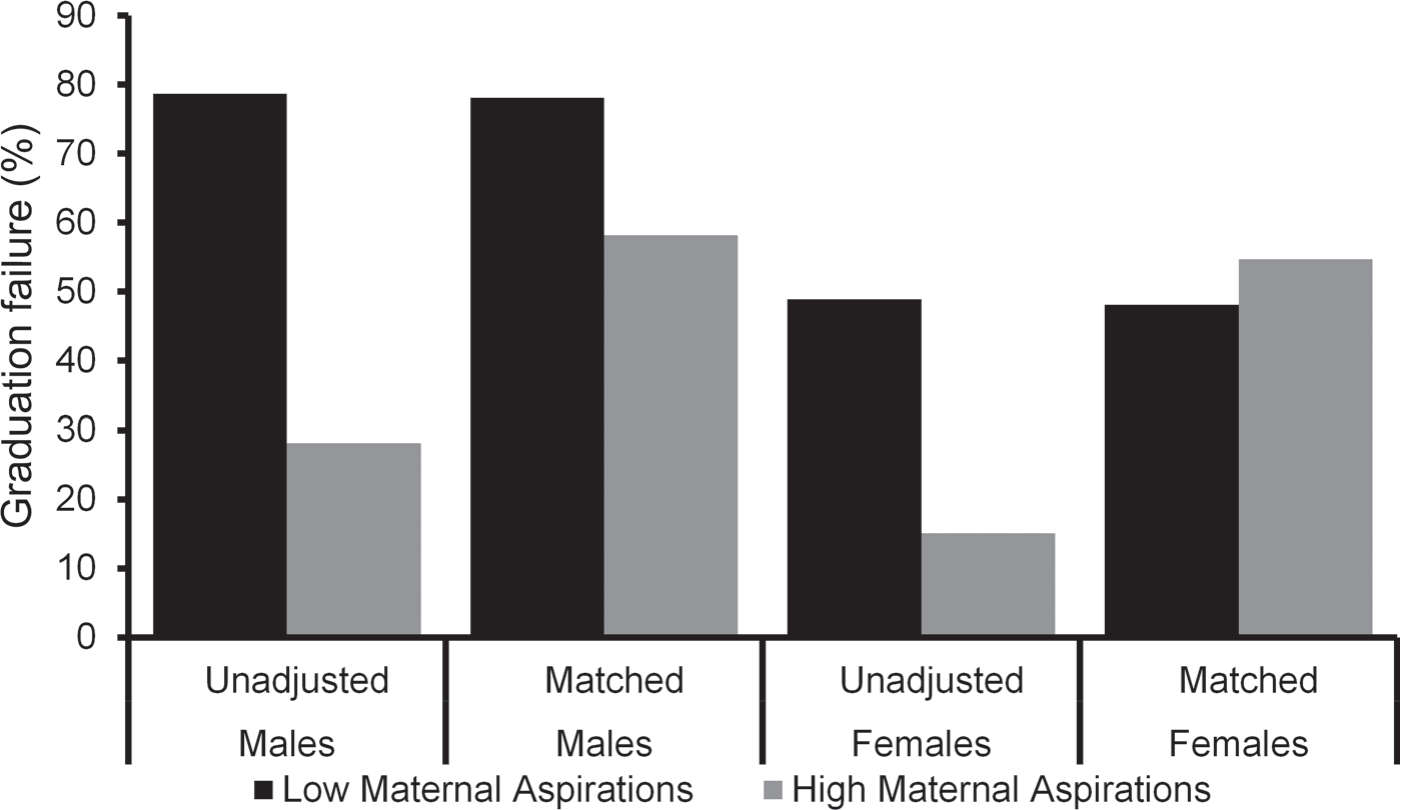
Predicted percentages of failure to graduate from high school according to sex and maternal expectations.

After matching, the risk ratio was further reduced compared to the fully adjusted model and was not statistically significant, RR = 1.16, 95% CI [0.94, 1.44]. However, in the matching model, sex and low maternal expectations interacted positively and significantly (p = .023) in the prediction of graduation failure, so that the analyses must take this interaction into account. To visualize the interaction, Fig. 1 presents the percentage of participants failing to graduate from high-school according to sex and maternal expectation status, using both the unadjusted and the propensity score matching models. A large majority of males experiencing low maternal expectations failed to graduate (78.8%), much more than for males experiencing high maternal expectations (28.1%), with a difference of more than 50% between the two groups. However, after matching, the rate of failure in those participants with high maternal expectations who were matched to participants with low maternal expectations was much higher (59.2%), showing that a large part but not all of the contribution of maternal expectations is due to confounding variables (see Fig. 1). For females, the difference between the two groups before matching was large (more than 30 percentage points) even though smaller than the difference for males. The difference was eliminated after matching, suggesting that the association of maternal expectations to graduation for females is entirely due to confounding variables. Table 4 presents the expected percentages and risk ratios as well as 95% CIs. The Matched Model row indicates that the contribution of maternal expectations after matching was still significant only for males.

**Table 4:** Risk Ratios and Expected Percentages of Graduation Failure According to Low/High Maternal Educational Expectations and Sex in Match Models.

		Males		Females	
		Expected values	95% CI	Expected values	95% CI
Unadjusted Model	Low Expectations (%)	78.8	[70.6, 85.3]	49.0	[38.9, 59.1]
	High Expectations (%)	28.1	[24.4, 32.1]	15.1	[12.3, 18.3]
	Risk Ratio	2.81	[2.38, 3.31]	3.26	[2.41, 4.30]
Matched Model	Low Expectations (%)	78.8	[70.8, 85.5]	48.8	[39.1, 58.8]
	*Matched* High Expectations (%)	59.1	[44.7, 71.7]	55.2	[40.0, 71.1]
	Risk Ratio	1.35	[1.07, 1.78]	0.90	[0.63, 1.28]
Matched Model with discard	Low Expectations (%)	77.2	[68.6, 84.3]	47.6	[37.6, 58.0]
	*Matched* High Expectations (%)	58.2	[44.0, 71.7]	52.3	[36.5, 68.3]
	Risk Ratio	1.35	[1.05, 1.78]	0.94	[0.64, 1.37]
Match Models for Boys and Girls Separately	Low Expectations (%)	77.2	[68.1, 84.7]	44.1	[33.9, 54.8]
	*Matched* High Expectations (%)	55.6	[41.4, 69.1]	40.0	[23.3, 57.4]
	Risk Ratio	1.42	[1.08, 1.89]	1.17	[0.70, 1.96]

*Note*. The expected values – percentages and risk ratios – in each sub-group are obtained from logistic regressions with 10000 simulations using an asymptotic normal approximation [46]. Confidence intervals (95% CI) are provided.

Finally, regarding sex differences in maternal educational expectation levels, mothers had somewhat lower educational expectations for their boys (18.5% of low expectations) than for their girls (14.5%), although the difference was only marginally significant (χ^2^ = 3.5, df = 1, p = .06). Conversely, clearly more males than females were not high-school graduates at age 22–23 years (37.5% vs 20.0%, χ^2^ = 47.0, df = 1, p<.001).

### Complementary analyses

#### Discard option

**Fig 2:**
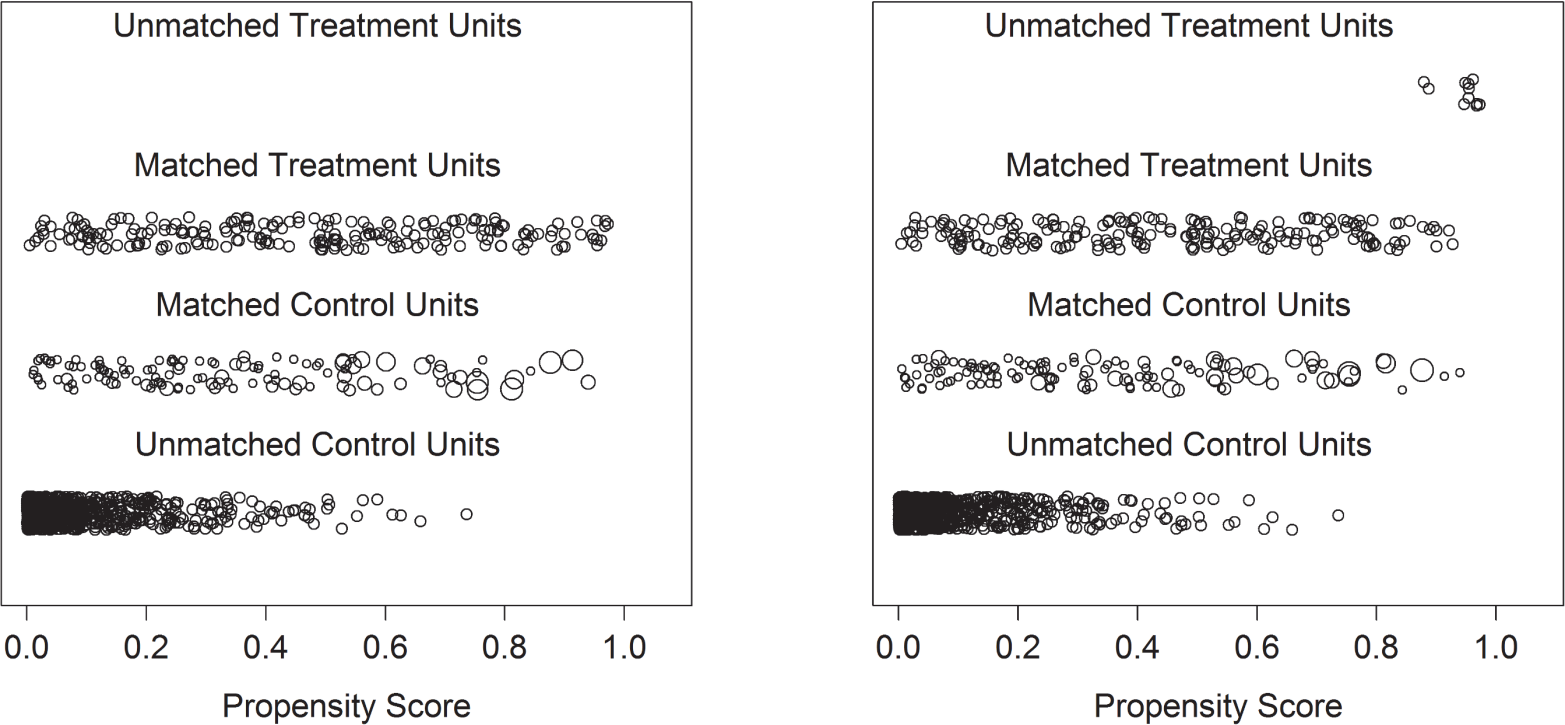
Distributions of the propensity score. Treatment Units correspond to children with low maternal expectations whereas Control Units correspond to children with high maternal expectations. The left part of the figure shows the overlap between the Matched Treatment Units versus the Matched Control Units. The Unmatched Control Units correspond to children with high maternal expectations who were discarded from the analyses (note the high density of children with low propensity scores in this group, meaning that, given their characteristics, these children had a very low probability of having a mother with low expectations and thus represented poor matches). The right part of the figure represents the matching procedure when the discard Treatment Units option was used (see *Discard option*, in the *Complementary analyses* section): some Matched Treatment Units had a propensity score close to one, which made it hard to find equivalent Controls. As such, they were discarded in this complementary analysis and are plotted as Unmatched Treatment Units.

As a complementary analysis, we tested the usefulness of discarding participants in the high and in the low expectation group who are outside common support (i.e. the overlapping range of propensity scores between the two groups). When we used this option, the distance was reduced from 0.037 to 0.021. The average number of controls used still remained 124 but, on average, 11 participants with low maternal expectations were discarded. The difference between the two procedures can be visualized in Fig. 2. The fact that only 124 controls were matched from the large pool of potential control children and that some children exposed to low maternal expectations were out of common support demonstrates that there was only a restricted overlap between the two groups. Therefore, estimating the role of expectations in children’s educational outcomes is subject to considerable selection bias. When we re-estimated the contribution of maternal expectations with the discard option, the interaction between sex and expectations was still significant; the risk ratios for males and females were virtually unchanged (see Table 4).

#### Separate analyses for males and females

Because the matching was done on the whole sample and not separately for males and females, it is possible that it was more efficient for one sex, thus introducing a bias in the results for the other one. We therefore re-run the last matching model (nearest neighbor with replacement and discard option) separately for males and females and re-estimated the results. Again, the contribution of maternal expectations was statistically significant for males but not for females (see Table 4).

## Discussion

The aim of this study was to verify whether low maternal educational expectations when the child was 12 years had a plausible causal contribution to graduation failure in early adulthood. Using propensity score matching to reduce confounding, we found that the contribution of low maternal expectations was not significant when males and females were considered together; when considered separately, its contribution was statistically significant for males but not females.

Whether educational expectations have a causal role in the prediction of educational attainment has been a central issue since the early literature on the topic [8]. Our results show that the strategy used in most of the available literature, i.e. adjusting for a restricted set of important confounding variables, does remove a large part of the bias but not all of it and, as such, may not accurately estimate the contribution of expectations to attainment. The results of this study are consistent with the possibility that extant research may have overestimated the contribution of parental expectations to educational attainment. Researchers might want to consider a wider range of potential confounding variables as well as use designs to strengthen causal inferences whenever possible. Reviews of these designs and statistical innovations are available elsewhere [10]. Each of these approaches has its strengths and limitations. For instance, although we included a large set of potential confounders, the existence of unobserved confounders is still possible (taking them into account would likely further reduce the role of maternal expectations). Therefore, although propensity score matching is utilized to minimize confounding it does not guarantee, by itself, a definitive evidence of the presence or the absence of a causal relationship. A comprehensive understanding of the causal role of parental expectations will stem from a convergence of findings from studies using different techniques and designs. In particular, successfully manipulating maternal expectations is crucial. However, a direct manipulation of maternal expectations is not possible as they cannot be randomly assigned to mothers. An alternative would be to: first, design an intervention that significantly impact maternal expectations; second, randomly assign mothers to the intervention and test whether the rise in maternal expectations translates into better educational outcomes; third, formally test whether maternal expectations mediate the impact of the intervention on educational outcomes.

It should be noted that our results do not necessarily apply to all contexts. Indeed, educational expectations are highly sensitive to historical and social contexts, which may also be the case for their effect on educational attainment. For instance, the role of educational expectations in the prediction of educational attainment seems to vary according to countries’ educational system [34]. In addition, following the sharp increase in youth educational expectations between the 1970s and the 1990s, the strength of the prediction of educational attainment by youth expectations dropped by half [48]. Similarly, the predictive role of parental expectations may have lessened with time.

### The role of sex

One of the most intriguing findings of this study was that maternal expectations made a statistically significant contribution for males but not for females when using propensity score matching. In this section, we discuss the relationships between sex, educational expectations and attainment.

The sex ratio for educational attainment has changed in favor of girls in recent decades [5]. The putative role of maternal expectations in explaining this difference between sexes in educational attainment can be twofold: 1) a difference between sexes in the levels of maternal expectations; 2) a stronger contribution of maternal expectations to educational attainment for girls. In this study, boys were more likely to experience graduation failure, which is consistent with the trend observed in recent decades [5]. Moreover, low maternal expectations were more common for boys but the difference was only marginally significant; this is in line with previous studies, which have reported no difference or higher parental expectations for females [6,12,16,49]. Therefore, it is unlikely that the sex difference in attainment is due to different levels of maternal expectations between sexes in the present study. Maternal expectations could have contributed to explain sex differences in attainment in another way: a stronger contribution of maternal expectations in females. However, after matching, maternal expectations made a significant statistical contribution to the failure to graduate from high school for males but not for females.

Very few studies have tested the interaction between sex and parental expectations in the prediction of educational attainment. In one study [29], parents’ expectations in 8th grade were associated with students’ own expectations in12th grade for boys more so than for girls. However, the same was not true for the contribution of parental expectations to achievement. Mello [28] reported that the association between youth occupational expectations at age 14 years and occupational attainment at 26 years was significantly higher for males than for females. However, the effect size was small and the same was not true for the association between educational expectations and educational attainment. Methods used may partly explain these discrepancies. Of significance is that we detected the interaction between sex and maternal expectations only when we used propensity score matching; the interaction term was also positive but not statistically significant in the unadjusted and the adjusted models. Another study also yielded similar findings with interactions being statistically significant only with the use of propensity score matching [50]. This suggests that, in the presence of a strong selection bias, regression techniques may conceal group differences.

Sex differences regarding home and school adjustment might explain why the contribution of maternal expectations was higher for boys in this study. Indeed, as compared to girls, boys seem to be more affected by stressful home conditions, and tend to have more school adjustment problems [1]. Girls are included in a wider peer network at school and educational expectations of their peers are more elevated [51]. Therefore, the lack of an association between high school graduation and maternal educational expectations for girls may be related to the fact that they are exposed to other sources of influences on educational attainment, like peers or teachers. Conversely boys may be more sensitive to low maternal expectations in the absence of buffering processes at school.

### Strengths and limitations

It should be noted that we used aspirations as a proxy of expectations. As discussed previously, both concepts have been shown to be highly correlated and have been used as proxies for one another [34]. However, other studies have reported some empirical differences between aspirations and expectations [9]. We were unable to verify to what extent both constructs differentially predict educational outcomes. However, we note that the item used in the present study was strongly predictive of the outcome when we controlled for a set of variables comparable to the one utilized in the literature. Therefore, the fact that no statistically significant contribution was detected when using propensity score matching (at least for girls) is unlikely due to the item we used. Our sample was fairly large but cannot be deemed as representative of the original population because of attrition, which is a common problem in long-term longitudinal studies. Without attrition, our sample size could have been larger, in particular for the low maternal expectation group, potentially increasing the possibility of detecting a significant role for maternal expectations. In addition, the propensity score matching procedure yielded a 1.16 risk ratio of graduation failure associated with low maternal expectations, which was not significant. Similar or even lower risk ratios may be significant in larger samples, leading to a somewhat different conclusion (i.e. low maternal expectations are significantly associated with a small increase in graduation failure).

Among the strengths of this study were the use of a long-term longitudinal design and measures from different informants (mothers, teachers and administrative data). In addition, to the best of our knowledge, it is also the first time that propensity score matching is used to minimize confounding when assessing the effect of maternal expectations on educational attainment.
